# Raising awareness of anti-fat stigma in healthcare through lived experience education: a continuing professional development pilot study

**DOI:** 10.1186/s12909-023-04889-8

**Published:** 2024-01-16

**Authors:** Christine Heidebrecht, Dianne Fierheller, Sara Martel, Alex Andrews, Amanda Hollahan, Laura Griffin, Sonia Meerai, Raeden Lock, Helia Nabavian, Chelsea D’Silva, May Friedman, Ian Zenlea

**Affiliations:** 1 Institute for Better Health, Trillium Health Partners, Mississauga, Canada; 2 Independent Scholar, Toronto, Canada; 3 School of Social Work, Toronto Metropolitan University, Toronto, Canada; 4 Faculty of Social Work, Wilfrid Laurier University, Brantford, Canada; 5 Program for Health System and Technology Evaluation, Ted Rogers Centre for Heart Research / Peter Munk Cardiac Centre, Toronto General Hospital Research Institute, Toronto, Canada; 6 Social Service Worker Program, Sheridan College, Oakville, Canada; 7 Postgraduate Medical Education, University of Toronto, Toronto, Canada; 8 Department of Women’s and Children’s Health Program, Trillium Health Partners, Mississauga, Canada; 9 Department of Paediatrics, University of Toronto, Toronto, Canada

**Keywords:** Anti-fat attitudes, Weight stigma, Weight bias, Lived experience education, Personal narrative, Co-design, Medical education, Continuing professional development

## Abstract

**Background:**

Anti-fat attitudes and weight-based discrimination are prevalent in healthcare settings and among healthcare practitioners and clinical trainees, and can result in immense harm to patients. There is increasing recognition that anti-fat bias in healthcare is a critical issue that must be addressed, but there is a dearth of evidence demonstrating sustained attitude and behavioural change among clinicians, illustrating a need for more innovative educational approaches and rigorous evaluation. We describe the co-design and delivery of a narrative-based continuing professional development curriculum aimed at raising awareness of weight-based bias and stigma.

**Methods:**

Our research team of lived experience educators, clinicians and researchers collaboratively developed a series of seven podcast episodes comprised of narrative descriptions of lived experiences with and impacts of weight bias, stigma and discrimination in healthcare settings, as well as a post-podcast workshop to facilitate reflection and discussion between participants. The curriculum was piloted among 20 clinicians practicing at a large urban hospital in Mississauga, Canada. We explored feasibility, acceptability and learning impact by analyzing responses to questionnaires completed following each podcast episode and responses shared during the workshops and follow-up feedback sessions.

**Results:**

We observed high acceptability and feasibility of the curriculum. Participants experienced the podcast as a practical and convenient learning format and the workshop as a valuable opportunity to collectively debrief and reflect. The learning impact of the curriculum was strong; participants described a range of emotions elicited by the podcasts, engaged in self-reflection, and expressed a desire to modify clinical approaches. Barriers to the application of learnings identified by participants include pervasiveness of the use of body mass index (BMI) as an indicator of risk and a criterion for referral; discomfort with difficult conversations; prevalent biomedical understandings about the association between weight and health; and clinicians’ defensiveness.

**Conclusion:**

This pilot study yielded promising findings and demonstrated potential impact on weight bias and stigma among healthcare providers. Necessary next steps include conducting larger scale, rigorous evaluations of the curriculum among broader populations, both health professions trainees and current healthcare providers.

## Background

Weight-based or anti-fat bias, stigma and discrimination are widely experienced by individuals living in larger bodies, collectively comprising an under-recognized system of social oppression [1–3]. Anti-fat biases are negative attitudes, beliefs and stereotypes about people based on their body size [4, 5]. These biases often result in weight-based stigma and the social devaluation of fat individuals, which can ultimately lead to weight-based discrimination [4, 5]. Globally pervasive [6] and often socially acceptable, anti-fat bias and discrimination occur across interpersonal and cultural contexts and societal institutions and can manifest as, for example, microaggressions – subtle, sometimes unintentional insults or displays of disrespect; ridicule and bullying; and differential treatment across many social spaces including in education, employment and healthcare [5, 7, 8]. As fatness intersects with other social identities such as race, gender, sexuality, and ability, weight-based bias, stigma, and discrimination can be amplified by experiences of oppression associated with an individual’s unique identities [7, 9, 10].

**Table:** 

***A note about language***
In this paper we use the word ‘fat.’ Many individuals and communities have reclaimed it as a source of empowerment and resistance and as a neutral descriptor rather than a derogatory term [11]. Moreover, we intentionally refrain from using the word ‘obese’ and its derivatives, which pathologize body size and project a value onto the person being described and their health status [12]. We agree with Fox et al., who assert that it is not possible “to simultaneously pathologize and destigmatize fat people” ([12] p.3). In addition to the word fat, we use the terms ‘larger body’ and ‘smaller body’ as descriptors

Individuals in larger bodies experience stigmatizing beliefs and discriminatory practices as chronic stress, resulting in significant physical, psychological, social, and financial impacts [13–17]. Weight stigma has been associated with an increased risk of chronic conditions such as arteriosclerosis, diabetes, and minor cardiac conditions [18]. Stigma and discrimination have also been linked to elevated blood sugar levels, which may increase the risk of type 2 diabetes and other biomarkers associated with cardiovascular disease and diabetes [15, 19, 20]. Moreover, the experience of weight bias and stigma can tremendously impact psychological health [15, 21]. Associations between weight stigma and depressive symptoms and anxiety disorders have been repeatedly documented [15, 21, 22]. Correlations between weight stigmatization, internalized weight bias, and disordered eating have also been widely observed [15, 19, 23–25].

Anti-fat attitudes and weight-based discrimination are prevalent in healthcare settings and among healthcare practitioners and clinical trainees [14, 26–30], and can result in immense harm to patients. Evidence demonstrates that clinicians treat patients living in larger bodies differently than those living in smaller bodies. Healthcare providers have been known to fat shame patients by blatantly expressing disgust [28] and using derogatory language or humour in medical settings [31]. They may spend less time with and perform fewer tests on larger patients [29, 32] and hold the perspective that patients in larger bodies will be less likely to follow care instructions [29]. Healthcare providers have been trained to practice within a weight-centered health paradigm that assumes a causal relationship exists between higher body weight and poor health, and that weight loss will result in improved health outcomes [33, 34]. Clinicians may assume that symptoms are caused by weight, and therefore weight loss might be recommended instead of performing clinical investigations [2, 35]. Further, healthcare spaces often lack inclusive furniture, medical equipment and/or clothing to support larger bodies, resulting in unwelcoming environments and barriers to accessing medical care [29, 36]. Stigmatization and discrimination within healthcare settings may result in feelings of stress and shame, and can negatively influence health-seeking behaviour, leading individuals to delay or avoid seeking care [29, 36–38] and potentially exacerbate the health impacts described above. Discriminatory clinician practices and/or altered health-seeking behaviours may result in delayed or missed diagnoses, and persistent recommendations of weight loss can increase the risk of disordered eating and result in weight cycling – repeatedly losing and gaining weight [33, 34]. Sustained weight loss is not possible for most individuals, and repeated attempts negatively impact cardiometabolic health [39–41].

There is increasing recognition that anti-fat bias in healthcare is a critical issue that must be addressed [42, 43]. Weight bias, stigma and discrimination were added to Canadian clinical practice guidelines for the first time in 2020 [44], and a small but growing body of literature has described interventions designed to reduce weight bias and stigma among healthcare providers and their impacts [45, 46]. However, there is a dearth of evidence demonstrating sustained attitude and behavioural change among clinicians, illustrating a need for more innovative educational approaches and rigorous evaluation [45, 46].

Personal narrative can play a meaningful role in medical and health professional education. Educational initiatives across multiple health disciplines and educational levels have incorporated patient stories and voices through videos, classroom lectures and personal interactions between patients and learners [47–52], demonstrating that narrative can foster empathy and compassion; reinforce the need for a holistic perspective; cultivate understanding that extends beyond biomedical knowledge; and challenge previously held assumptions and beliefs about a condition or patient group [47–49, 53]. Studies have shown that empathy is negatively associated with prejudice [54, 55] and can lead to more affirmative attitudes about stigmatized groups [56]; accordingly, pedagogical tools that evoke empathy, such as personal narrative, may be particularly valuable when striving to reduce bias and stigma.

Lived experience education (LEE) is an approach to education that is informed by individuals who have experienced moving through the world with a certain identity or as a member of a particular community and is one way to incorporate narrative into educational initiatives. The incorporation of LEE in research and training is supported by a growing literature on co-design approaches in mental health and healthcare, which acknowledge lived experience as a valuable form of knowledge and expertise that should be centred in healthcare research, design, and advocacy [57–62].

The project described in this paper was a collaboration between researchers, clinicians, and lived experience educators, and focused on co-designing educational materials which incorporated educators’ narratives as part of a professional development curriculum to raise awareness of weight-based bias and stigma among healthcare providers and trainees. These narratives were developed into a series of podcast episodes designed to be accessed by and delivered to clinical learners asynchronously, followed by a group workshop.

To explore feasibility and acceptability, we invited hospital-based clinicians to engage with the podcasts, provide written feedback, and participate in a one-hour workshop to discuss the content and their learning. In this paper, we describe the curriculum development process and observations of the curriculum’s acceptability and learning impacts as a continued professional development tool. We also identify and explore some of the barriers to the incorporation of learnings into clinical practice.

## Methods

### Setting

This pilot study was conducted by researchers based at the Institute for Better Health (IBH) within Trillium Health Partners (THP), a teaching hospital in Mississauga, Canada; York University; and Toronto Metropolitan University in Toronto, Canada. The study was conducted with clinicians within THP’s Women’s and Children’s Program. Ethics approval was granted by the research ethics boards of THP and Toronto Metropolitan University. All lived experience educators and clinician participants provided informed consent prior to the initiation of study activities.

### Participants

#### Lived experience educators

Lived experience educators (henceforth described as ‘educators’) were invited to participate via email through student listservs within programs to which one of the researchers was connected: Toronto Metropolitan University’s School of Social Work and York University’s Graduate Program in Gender and Women’s studies. This invitation was not posted by the researcher connected with these programs to mitigate the risk of perceived coercion. Recruitment materials included contact information for this researcher and project leads who were not affiliated with either university, to allow prospective participants to choose who they felt most comfortable contacting. The consent process was carried out by a team member not affiliated with either university. Individuals 18 years of age or older who self-identified as fat, had experienced weight-based stigma and discrimination in healthcare settings, and were willing to share their experiences, were eligible to participate. Educators played a unique dual role as both research team members and participants. They worked alongside the research team to co-create study materials and participated in data analysis while also being asked to share responses and feedback about study processes. Four individuals consented to participate as educators, all who were students (two undergraduate and two graduate). A fifth individual, also a student, consented to participate but withdrew in the planning stage due to other time commitments. Resources available to support educators’ emotional safety throughout the study included multiple social workers on the research team, at least one of whom was present at each meeting and whom educators could contact outside of meetings, as well as a list of local mental health supports. Educators were provided with honoraria to compensate them for their time on study activities.

#### Clinician participants

Clinicians from any discipline practicing within THP’s Women’s and Children’s Program were eligible to participate as recipients of the curriculum. Email invitations were disseminated to all staff members within the program, approximately 620 individuals. Among these, 4% were midwives, 4% were allied health professionals (occupational therapists, physiotherapists, dieticians, social workers), 16% were physicians and 76% were nurses. Clinicians were provided with gift cards as an expression of gratitude for their participation.

### Curriculum development

The combination of podcasts and a workshop as learning platforms was chosen for multiple reasons. Podcasts are increasingly used as educational tools across various clinical disciplines and academic levels [63–68]. They are regarded positively by medical learners who value the convenience, the option to repeat content to consolidate learning, and the aural nature of the content [64, 67, 69]. Podcasts may also enhance learners’ perceived connectedness to educators [67]. The workshop, facilitated by social workers with clinical, research and lived expertise related to weight stigma, was designed to be a reflective space for professionals to probe their attitudes and practices related to weight and health, learn with and from peers, and explore strategies for enacting these learnings in practice.

We wanted the educators’ knowledge and lived experiences, recommendations and identified priority areas to guide the content of the podcasts. Therefore, we began the planning process alongside the educators with no a priori ideas about podcast content or format. We brought together study investigators and educators in a series of in-person meetings: an initial meeting for introductions and early brainstorming, followed by two planning meetings during which ideas about podcast content and presentation were exchanged and discussed. A set of episode outlines and potential discussion prompts were drafted based on these meetings and shared with educators for feedback prior to being used as a conversation guide during three recording sessions. This guide was comprised of open-ended questions designed to engage educators in dialogue about their experiences in healthcare settings. Recording sessions were initially scheduled to take place in person in the spring of 2020, but due to COVID-19 pandemic restrictions, we modified our approach and held recording sessions over Zoom. To foster an environment of emotional comfort and safety, only educators and a facilitator – one of the study investigators who also lives in a larger body – were present during recording sessions. An unrecorded debrief session was held immediately following each recording session. Educators were sent a transcript of each conversation in which they had participated so that they could review and classify content as “exclude,” “definitely include,” “could include,” and “not that valuable.” Transcripts were drafted into episode outlines which were sent to all educators for review and then edited into podcast episodes that all team members also reviewed prior to finalizing. The seven episodes comprised introductions to the topic of weight stigma, language, and the educators; narrative descriptions of lived experiences with and impacts of weight bias, stigma and discrimination in healthcare settings; and recommendations for clinicians about how to address this oppression (see Table 1). Episodes ranged in length from 8 to 21 min. A supplementary resource page containing summary and reflection points and references to support further learning, was also developed to accompany each podcast episode (see Appendix 1).

**Table 1:** Podcast episodes titles and summary points

**1. Introduction to the podcast:** *“By undervaluing weight stigma, clinicians are perpetuating harm.”*
▪ Like other forms of systemic oppression, anti-fat bias harms people across the life span and can lead to chronic stress and significant impacts on health and wellness
▪ Sustained weight loss is unachievable for the vast majority of individuals; by focusing on weight loss, clinicians are perpetuating a culture of eating distress
▪ An individual’s unique social identity includes categories such as race, gender, and sexual identity, all of which intersect with fatness. Weight-based stigma can heighten experiences of oppression at these intersections
▪ Fat people deserve to be seen, heard, and respected as human beings, and for their health and wellness to be considered outside of the context of body weight
**2. Introduction to speakers and language:** “*I love that there is power, that I can take power back by using that word.”*
▪ Treatment experiences in healthcare settings for individuals living in larger bodies often intersect with body weight, health behaviours, and feelings of shame
▪ Words often used by medical professionals (e.g. “overweight,” “obese”) can be experienced as violent, othering, and deeply harmful
▪ Each patient will have language they prefer and language they don’t. The best practice is to ask them what feels right to them and follow their lead
▪ For example, the word “fat” has been reclaimed by many individuals and communities as a source of empowerment and resistance and is a neutral descriptor rather than a derogatory term. However, not everyone living in larger bodies is comfortable using this word
**3. Lived experiences I:** “*Regardless of what I go in for, I leave feeling like my body is wrong.”*
▪ Healthcare providers commonly centre appointments around body weight and weight loss regardless of the reason for the medical visit
▪ Unnecessary focus on weight in an appointment can elicit feelings of shame and discourage people from seeking health care
▪ Experiencing care and respect from medical professionals can be rare
▪ When healthcare is sought, numerous additional barriers impede access and meaningful treatment
▪ Considerable emotional labour is required to prepare for, participate in, and recover from medical appointments
**4. Lived experiences II:** “*You are the problem…and that’s why you can’t do this thing.”*
▪ Fertility challenges experienced by the educator were immediately connected to body weight by medical practitioners
▪ The language a clinician uses is very powerful; it can invoke fear, shame and guilt and silence a patient’s voice and ability to self-advocate
▪ The messages the educator receives about herself at every medical appointment are harmful to her overall mental health; immense amounts of time and emotional labour are required to recover following appointments
**5. Health impacts of weight stigma:** *“You would be very surprised to know how many fat folks walk out of a doctor’s appointment feeling like absolute shit.”*
▪ Diagnoses among fat patients can be delayed or missed altogether because symptoms are often attributed to body weight
▪ A lack of furniture, equipment, and clothing that comfortably fit larger bodies can be dehumanizing and traumatizing
▪ Medical appointments that primarily focus on body weight leave fat patients feeling blamed, shamed, and scared to return
▪ The stress of weight stigma takes an incredible toll on fat patients’ health
▪ Empirical measurements (e.g. blood pressure, blood sugar) may be influenced by anxiety and fear experienced in anticipation of criticism and shame during a medical appointment
**6. Distressed and disordered eating:** *“Your weight is going to determine how you’re treated, versus your actual needs.”*
▪ Many individuals who experience disordered eating identify interactions or interventions within the medical system – often as early as childhood – as being at the root of their eating distress
▪ Disordered eating is often not recognized in fat patients, who are praised for losing weight and becoming “healthy”
▪ When disordered eating is diagnosed among individuals in larger bodies, the treatment received and support available can differ substantially from those received by individuals in smaller bodies
▪ Intersecting social identities and weight-based discrimination can compound eating distress; one educator describes the incompatibility between the criteria for gender-affirming surgery and recovery from disordered eating
**7. How can clinicians do better:** *“Give control and self-determination to us, to make our own choices on how we want to speak on our bodies.”*
▪ Be ok with not knowing all the answers
▪ Get comfortable having uncomfortable conversations; ask patients if they would like to talk about body weight and, if yes, what language they’d prefer to use. Listen to and respect their wishes
▪ Familiarize yourself with the current evidence about weight and health
▪ Familiarize yourself with the potential harms associated with a weight-focused approach and prescribing weight loss as an intervention
▪ If you think it is necessary and relevant that a patient’s weight be discussed, ask their permission
▪ Ensure that all body sizes are represented in medical education and training
▪ Ensure that your practice includes medical equipment, clothing and furniture that fit all body sizes

The post-podcast reflection workshop for clinician participants was also collaboratively designed by the research team. The format and discussion questions were discussed at a planning meeting to which all members of the research team, including educators, were invited. A draft discussion guide was developed and circulated electronically to all team members for review and comment.

### Implementation and data collection

Podcast episodes, accompanying resource pages and survey links were provided to clinician participants several weeks in advance of the reflection workshops. Two workshop dates were offered to accommodate participant schedules. These materials were made available both on a secure study site on THP’s server as well as through a secure web-based file sharing platform. Participants were asked to complete a demographic survey prior to listening to the podcasts and complete a post-podcast survey following each episode. The survey included open-ended questions inviting respondents to share their immediate reflections or responses to the episode, describe the content that was most and not important/relevant to their clinical practice, and identify topics which they wished were included and/or expanded upon. Likert scale questions asked about the usefulness of the delivery approach, sound quality and appropriateness of each podcast episode’s length.

Clinician participants then attended one of two 60-min reflection workshops, which were held over Zoom and facilitated by members of the research team. Participants were asked to introduce themselves and describe what brought them to the project as well as their reactions to the podcast materials. Participants were then asked about what they learned, whether these learnings had informed their clinical practice, perceived barriers to incorporating these learnings into healthcare spaces, and next steps for the work. To foster participant safety, the workshops were not recorded, but notes were taken. Immediately following each workshop, another research team member facilitated a post-workshop feedback session during which participants were asked to share thoughts about the content and delivery of the curriculum, what could be improved, and whether they would recommend the curriculum to colleagues. Individual feedback sessions were held with participants who either could not attend the post-workshop feedback sessions, preferred to share feedback privately or were unable to participate in the workshop. These sessions were recorded, and notes were made from the recordings.

### Data analysis

Clinicians’ open-ended survey responses and workshop and feedback session notes were analyzed thematically following Braun and Clarke’s six analysis phases [70], applying a participatory approach. Educators were invited to participate in the analysis process as an optional add-on to their participation in the curriculum development and were provided with additional honoraria for compensation. Based on a modified version of DEPICT model for collaborative qualitative analysis [71], the analysis approach was designed to facilitate educators’ engagement in the capacity that best aligned with their availability and emotional energy. Two educators, who had experience with qualitative research, chose to participate. The analysis team was comprised of these educators as well as two THP researchers. After team members had familiarized themselves with the raw data, the group met over Zoom and collaboratively coded a sample of the meeting notes and open-ended survey responses. These codes were developed into a codebook, reviewed by, and corroborated with all team members before it was applied to all the data. Team members also reviewed all coded data to ensure that codes had been applied appropriately. The analysis team then came together for a series of three analysis meetings, before each of which code reports were circulated for review together with questions to reflect on while considering categories and themes [71]. The educators were invited to engage in this preparatory work, participate in analysis meetings, or both. At each analysis meeting, themes, sub-themes, and relationships were discussed and incorporated into an inventory of themes and corresponding notes, which was modified iteratively until all data were reviewed and consensus around themes among the team was reached.

## Results

### Participant recruitment and attrition

**Fig. 1:**
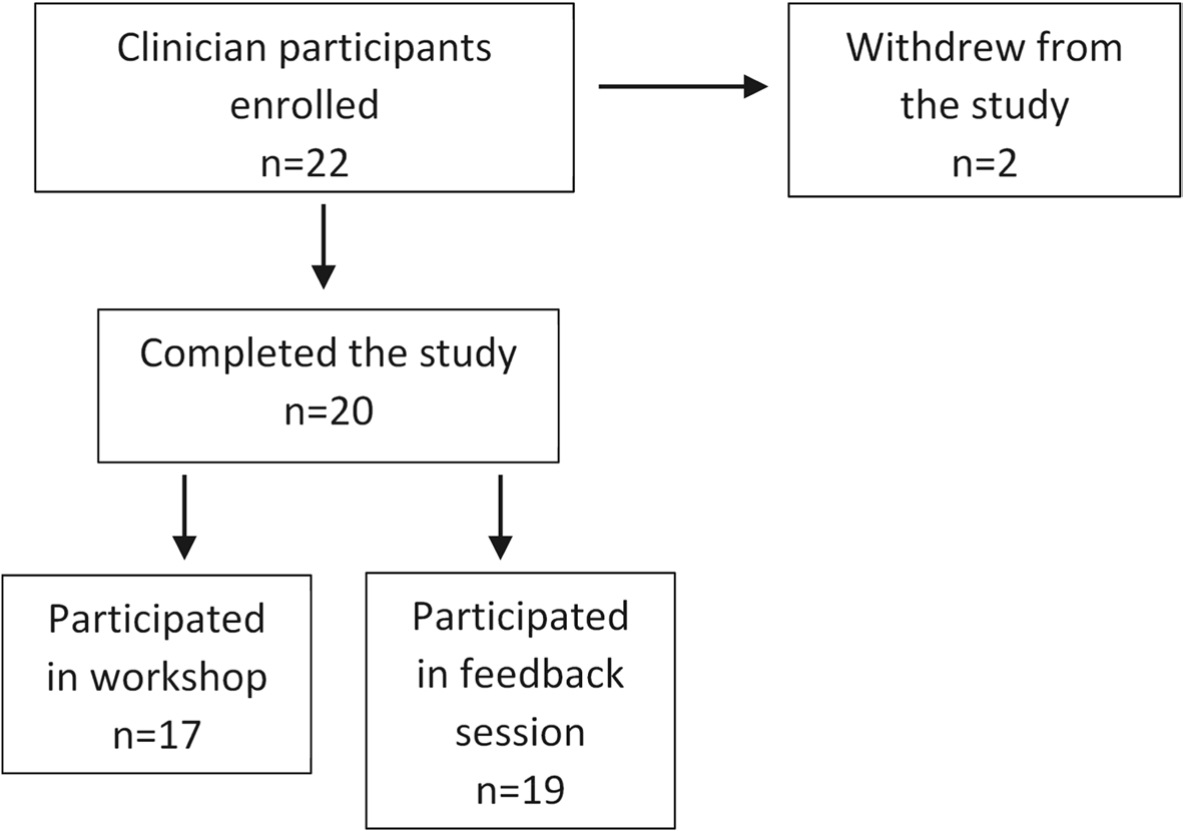
Participant enrollment, withdrawal and study completion

Twenty-two clinician participants were recruited, and two withdrew before the workshops were held. One participant withdrew because they did not feel they could participate objectively due to their personal views about an association between weight and health challenges, and a second withdrew due to a lack of time to participate in study activities. All but three of the remaining 20 clinicians participated in one of two reflective workshops. The three that could not attend participated in a post-workshop feedback session; in total, the post-workshop feedback sessions were attended by 19 participants (Fig. 1). Eighteen clinician participants completed the demographic survey, and 15–19 completed the survey that accompanied each podcast. Participants’ demographic characteristics are described in Table 2.

**Table 2:** Demographic characteristics^a^ of clinician participants (*n* = 18)

Characteristic		Frequency (%)
Gender identity	Female	18 (100)
Age (years)	26–35	3 (16.7)
	36–45	6 (33.3)
	46–55	6 (33.3)
	56–65	3 (16.7)
Ethnic or cultural identity	South Asian	1 (5.6)
	East Asian	1 (5.6)
	Canadian	2 (11.1)
	White	10 (55.6)
	Multiple identities	4 (22.2)
Professional role	Physician	3 (16.7)
	Registered nurse	4 (22.2)
	Midwife	5 (27.8)
	Allied health professional	6 (33.3)
Number of years in role	≤ 5	2 (11.1)
	6–10	2 (11.1)
	11–20	10 (55.6)
	> 20	4 (22.2)

^a^All demographic questions contained in the survey were open-ended; respondents self-identified gender and ethnic or cultural identity/ies. For the purposes of this table, racial categories from Ontario’s Data Standards for the Identification and Monitoring of Systemic Racism [72] were used, together with “Canadian”, a self-identified term, and “Multiple identities”, which was used when more than one ethnic or cultural identity was shared

Most clinicians (78%) indicated that they were moderately aware of weight bias and stigma in healthcare settings, while three (17%) described being very aware, and one (6%) reported that they were unaware. Half of the respondents described having previously participated in learning about weight-based bias and stigma, including in their undergraduate or graduate education, as professional development and/or on their own time.

### Acceptability and feasibility

We observed high acceptability of the curriculum format. When asked in the post-podcast surveys about the extent to which they agreed with the statement,* “I found the way that the information was delivered to be useful for learning about people’s experiences with weight bias and stigma,”* most respondents agreed (see Appendix 2). Ratings of “Strongly agree” and “Agree” were given by 84%-100% of respondents across the seven podcast episodes. The proportion of participants who indicated “Neither agree nor disagree” and “Disagree” was marginally higher in the surveys corresponding to the first two episodes. Most participants also perceived that the length of each podcast episode was appropriate. Participants appreciated the opportunity to debrief and reflect on the content as a group and the ability to listen to the podcasts on their own time and at their own pace (and to re-listen to content as desired). However, it was suggested that convenience could be increased by making materials available on mobile devices, and multiple participants said they would have liked more than the allotted hour for this post-podcast dialogue. Some clinicians pointed out that scaling the intervention to a broader range of learners may be challenging, given the time required to complete the curriculum. The high level of acceptability, the rate of completion, ease of dissemination of the curriculum materials, and the number of participants who recommended that the materials be made available to a broader range of health practitioners demonstrate the initiative’s high feasibility.

### Themes observed within clinician responses

#### The learning impact of the curriculum

Within open-ended survey responses, the learning impact of the curriculum was described as strong. Clinicians shared the power of hearing patients’ lived experiences and reported a range of reactions and responses to the podcast content. Many participants expressed anger, sadness, dismay, and surprise about educators' treatment in healthcare spaces.

*Wow! That was an incredibly powerful episode and very though provoking. The opening remarks about the word fat ("a body variation based on size") really stuck with me. I listened to it several times.*

*Shocked, disgusted, dismayed....that someone cannot receive gender affirming surgery due to BMI. Can't be their authentic self until they become someone different (lose weight).*

*It is very sad to hear how harmful fat people find health care appointments to be. My heart aches for those who find appointments so emotionally draining (even those as short as 15 minutes).*

*How the conversation is framed is soo important. I am flabbergasted at the energy that this person needs to attend their own appointment- gives me anxiety even listening.*

*My heart was breaking for the experiences of this individual. I work in the area of reproductive health and also had negative experiences myself as a fat patient. The impact of language, how we say something and the words we use, is so important to create a sense of trust and safety and greater emphasis needs to be placed on this in all areas of health care.*

In both the reflective workshops and survey responses, several clinicians described engaging in self-reflection about personal experiences of weight stigma, thin privilege, and the power and privilege that healthcare providers possess, as they listened to the content.

*My own bias and the awareness that my thoughts sometimes go towards: why don't they just lose weight?*

*Further reflection on my own bias and how these go beyond weight but into areas such as culture. Also recognition of the problems with our problem-based health care system where you have a problem and see someone about that specific problem but the focus is often not on the whole person.*

*Empowering in the sense that I am a fat person so it was refreshing to hear that my negative experiences with health practitioners are valid and real.*

*I'm reminded of the privilege we have as physicians interacting with patients and what a responsibility that is. Words matter. I found the statement highlighting that for us it's a 10- minute appointment, but for the patient it is a much longer experience* [to be impactful]*.*

*As a person with thin privilege, I do not experience this barrier with accessing care - something I have taken for granted. In fact, in the past, I have been explicitly praised for my "healthy" body before any objective medical assessments have been made- speaks to the biases within healthcare workers.*

Table 3 summarizes the key learnings and takeaways from the curriculum that participants identified through the workshops, feedback sessions, and survey responses. These encompass new understandings and areas of renewed awareness.

**Table 3:** Clinicians’ key learnings

Weight stigma during healthcare interactions can negatively impact health seeking behaviour and quality of care received; cause emotional distress, requiring preparation for and recovery from medical appointments; result in lasting impacts of ‘minor’ clinical interactions, and disordered and distressed eating
Language is important and words are powerful
Anti-fat stigma is oppression and a social determinant of health
There is a lack of inclusivity within the healthcare system which negatively impact access to care, care delivery and experiences, and representation of fat bodies
It is important to take a whole person approach to care
Appointments should be centered on patients’ priorities

#### Desire for lived experiences to be supplemented with biomedical evidence related to weight and health

Although, as outlined above, most participants appreciated the role of personal narrative in their learning while engaging the podcasts, a small number of participants raised concerns about the lack of content in the curriculum about the relationship between weight and health, and expressed a desire for biomedical evidence related to weight and health and weight stigma to be shared alongside lived experiences.

*The way physicians think is really evidence-based, scientific. Really helpful if very specific evidence was articulated. e.g. delayed diagnosis, missed diagnosis, a loss of trust in that physician because the reason I came to the office was completely ignored. People will then recognize that in their own experiences.*

*The speaker talked about "myths" related to weight being a determinant of health, it would be good to expand on that and give more evidence to support it as that would probably be more powerful in shifting people's thinking.*

#### Feelings of shame and defensiveness

In survey responses, workshop discussions and feedback sessions, a smaller number of respondents described feeling shamed by or defensive about the claims made by the educators in the podcasts or anticipated that future listeners might experience these feelings. Some respondents identified that some of the treatment described in the podcasts was due to systemic constraints (e.g. a lack of time to get to know a patient more holistically), while their intention was not to cause harm.

*There also seems to be an undertone of assuming (or accusing) all care providers or people have this bias; sounded slightly shaming. Keeping in mind that most healthcare providers are caring people (hence in the caring profession) and that a lot of the "biases" may be a consequence of the connection between weight and health that we've all been learning.*

*My only worry with us expressing defensiveness, is that it's justifying our behaviours. The reality is, healthcare isn't perfect; we can blame the government, we can blame somebody else, but admitting our time constraints is one thing* [referring to comments in workshop]*, but we can't just leave it at justification, we have to do more to try to change it. It's easy to feel defensive, and I felt defensive at times too - because I need weight to do a referral - but how do we break down that defensiveness?*

Skepticism about the validity of some lived experiences shared in the podcasts was also expressed through survey responses questioning the violence of terms such as “overweight” and the responsibility attributed to some healthcare providers for disordered and distressed eating.

*The resource sheet indicates "Words often used by medical professionals (e.g. “overweight”, “obese”) are experienced as violent, othering, and deeply harmful." Can we be that emphatic and certain in this assertion? That using the term "overweight" is violent? As with all things in medicine, nothing is 100%, so I think it would be important to bring in other viewpoints from people living in larger bodies.*

Some clinicians felt that the tone of some of the messages communicated – i.e., that could make clinicians feel defensive or attacked – could be modified to reach and facilitate the learning of as many healthcare providers as possible, though some appreciated that the content shouldn’t be modified just to make listeners comfortable.

*Whenever there are shifting things in our society, the best chance for buy-in is to have it introduced in a less aggressive way.*

*I am not sure that the comment made by one of the educators is useful and should be included:* "I want healthcare providers to feel bad. I want them to sit in that. I want them to feel the years and years of damage..." *I think that by listening to these stories, healthcare providers will get the message. It's not by making someone "feel bad" that you will get positive change.*

*I personally appreciated the honesty of the podcast and I understood it as coming from these individuals' experiences - and as I can identify with them and have wanted to share my own voice in this area, but I think that people could stop listening because they're feeling shut down, and defensive* ***.*** *Perhaps somehow an acknowledgement that these can be challenging conversations, they can result in feelings of defensiveness, and that's not the intent - the intent is about broadening perspectives and knowledge bases.*

#### A desire to change clinical practice

A desire to modify clinical approaches and provide better patient care – as well as to receive practical guidance about how to do so – was also expressed in survey responses and feedback sessions.

*This podcast makes me want to do better as a clinician.*

*How I can make 'small' changes (i.e. gown size, chairs, etc) that communicate care and value; consistently take the time to answer any questions/concerns that patients may have; create opportunities for dialogue re: experience and how I can better support patients.*

*I had not considered the importance of visual representation of different sized bodies in my clinical space. I will definitely be mindful of representation moving forward.*

*Recognizing how my reaction, or the anticipation of my reaction, influences the time we spend together both clinically and personally; that the whole medical culture has to change in order for there to be an appreciable difference*

*My role as a practitioner working directly with patients. How do I perpetuate these biases/stigma and what harm am I inadvertently doing to patients both emotionally and physically due to my own ignorance? Reflecting seriously on my own biases and what I can do to change the experiences of patients.*

*Important to not put the burden of education, and how to put it into practice, onto the people who have been traumatized by their experiences. Isn't it my job* [as a clinician]* to figure out what I can do? I need to take responsibility for that.*

#### Curriculum feedback

Participants expressed broad consensus regarding the need to increase awareness of and challenge weight bias and stigma within the healthcare system. Participants provided curriculum-specific feedback to improve its impact and support awareness raising; in addition to suggestions related to length and organization, broader content recommendations were also made in feedback sessions and survey responses.

Many participants described the value of additional practical guidance and recommendations about specific actions that could be taken with respect to developing a more weight-inclusive practice so that they would be better equipped to apply the learnings.

*As a clinician I am always keen to hear specific recommendations, including scripts for how to change.*

Clinicians also identified barriers that may impede their or other clinicians’ ability to apply learnings about weight bias and stigma to practice: the pervasiveness of the use of body mass index (BMI) as an indicator of risk and a criterion for referral (about which multiple clinicians expressed frustration); discomfort with difficult conversations; prevalent biomedical understandings about the association weight and health; and clinicians’ defensiveness.

## Discussion

In this pilot study, we observed high feasibility and acceptability of our novel curriculum designed to raise awareness of weight bias and stigma in healthcare settings among clinicians practicing at a large urban hospital. Participants shared feedback about the convenience of the podcast medium, and reported that the mode of delivery was useful for learning about weight-based bias and stigma. All participants described the curriculum content as impactful and reported new learnings and renewed understandings of the harms of weight stigma.

While almost all clinician participants entered the study with an existing awareness of weight bias and stigma in healthcare, the magnitude of the weight-based oppression experienced in healthcare settings – particularly the depth of its emotional, physical, and psychological impact – was surprising to many, demonstrating the need for more comprehensive education even among those who are aware of the problem. Reactions included shock, anger, disgust, and sadness about the experiences that fat patients have had in healthcare settings. Interestingly, participants who lived in larger bodies also expressed that some of the educators’ experiences mirrored their own as patients. Our findings are supported by previous studies that demonstrate the impact that patients’ voices and narratives can have on empathy, recognition of the importance of holism in care, and reflection on clinical practice among health professions learners when integrated into educational initiatives [47, 48, 50–53, 73].

Several participants in our study described discomfort and uncertainty about how to modify their practices appropriately, some expressed defensiveness, and a small number suggested that the curriculum’s receptivity might be increased if the tone of the podcast material was less critical of clinicians. Drawing similarities to some of the elements of white fragility [74], one team member (AH) aptly described these reactions by people in a position of power as indicative of “provider fragility.” Kumagai [53] posits that through the full range of emotional responses they evoke, patients’ stories can impart meaning to learners and can also create cognitive disequilibrium by presenting new or unfamiliar ideas that “challenge the validity of one’s worldview”(53 p. 656); this disequilibrium can be a catalyst for self-reflection and can ultimately result in a more expansive worldview [53]. The incorporation of lived experiences and patient narrative within educational interventions has the potential to be transformative for issues such as weight bias. Still, it must be done in a way that recognizes the potential for and addresses defensiveness and fragility.

While many participants found the narratives alone compelling, a small number of clinicians recommended that we heighten the impact of the curriculum by supplementing lived experiences with scientific evidence. It may indeed be beneficial for educational interventions aimed at addressing weight bias and stigma among healthcare providers and trainees to present evidence about the harms of a weight-centered health paradigm [33, 40, 75, 76], and the benefits of a weight-inclusive approach to healthcare [77, 78] – for example, Health at Every Size (HAES®) [79] – alongside lived knowledge. In addition, clinicians expressed a desire to have more practical guidance around actions that could be taken to develop a more weight-inclusive practice, signalling that the curriculum provided persuasive information about “why” to address weight stigma but could benefit from more in-depth content to guide the “how.”

Together with participants’ remarks about prevalent biomedical understandings regarding weight and health, and in particular, the pervasiveness of BMI as an indicator of health and risk, the observations about evidence described above prompted discussions throughout the analysis process about whose knowledge is valued and prioritized and whose definitions of risk and harm are valued and prioritized. Evidence-based medicine has traditionally adhered to a knowledge hierarchy in which narrative and lived experience are low and quantitative findings from meta-analyses of randomized controlled trials are at the pinnacle [80–82]. In recent years, however, scholars have drawn attention to knowledge inadequacies within medicine and have advocated for a broader approach to evidence-based medicine [80, 82, 83]. Beames et al. [83] argue for incorporating lived experience within integrated reviews and data syntheses to inform the development of clinical practices that are aligned with the priorities of patient populations. Greenhalgh et al. [80] assert that data produced from research that centres and explores the voices of those with lived experience provides meaningful context to statistical observations and “should be viewed as complementary rather than inferior to epidemiological evidence”(80 p.3). Lastly, Dahl-Michelsen et al. [82] have proposed an *Inclusive Evidence-Based Practice Model* in which clinicians’ professional praxis comprises three intersecting and dynamic circles: research-based knowledge; ethical care and experience-based knowledge; and patient knowledge and user involvement, centred within a larger circle of context. We support these calls for broadening evidence within research and medical education and suggest that it may help make current and future healthcare practitioners receptive to learning from patients’ lived experiences.

Safety, trust, and power are critical in research conducted alongside patients and communities. We endeavoured to foster environments of emotional safety and trust for educators by being intentional about who was present during podcast planning meetings (all members of the team except the Principal Investigator, who is a physician and has worked in pediatric weight management spaces) and recording sessions (only those with lived experiences); consistently providing opportunities to debrief; and ensuring that no content was shared without educators’ review, opportunity to provide feedback and approval. However, by not including all team members in, at minimum, the introductory meeting, a separation between the educators and the PI, and thereby the community and medicine/academia, was created, which may have been exacerbated by the language that was used, e.g. using ‘researchers’ to describe some team members, implying that others were not researchers. Further, there were disparities in lived experience between the educators and the rest of the research team, all of whom live in smaller bodies except for one investigator. It would have been valuable and perhaps contributed to a flattened or flatter hierarchy across team members [84], for us to have had an open conversation as a whole team at the outset of the study about knowledge, power, language and positionality, and how we could work together to mitigate imbalances. Educators were invited to submit anonymous memos throughout the study to share their experiences, and while they did feel that a safe space had been created, concerns about these other areas were identified. Educators shared additional reflections in a chapter in an edited collection of works about weight bias in health education co-written by the team [85]. However, in the future, it would be beneficial to provide educators with opportunities to share their feedback about power, safety, and trust unreservedly, perhaps through conversation or an interview with an individual unconnected to the study.

There are additional limitations of this study that are important to identify. As this was a pilot study designed to explore feasibility and acceptability, our sample size was relatively small, and we did not use psychometric tools or open-ended interviews to facilitate an in-depth evaluation of the impact of the intervention, including potential impacts on clinical practice or patient care. Further, as this was a voluntary educational intervention, our observations may have been impacted by self-selection bias; clinicians who chose to participate may have been more aware of weight bias and stigma as a challenge that needs to be addressed within healthcare. We did not have a full range of perspectives across gender or race and ethnicity within lived experience educators or clinicians; educators identified as women and non-binary, all clinician participants identified as female, and most of both groups were White. Lastly, because this study was conducted during the COVID-19 pandemic, almost all study activities took place over Zoom rather than in person. This may have impacted rapport and trust among educators and/or clinicians and, consequently, the experiences and reactions they were comfortable sharing.

## Conclusion

Our pilot study of a novel continuing educational curriculum co-designed with and featuring the narratives of lived experience educators yielded promising findings and demonstrated the potential impact on anti-fat bias and stigma among healthcare providers. Important next steps include conducting larger scale, rigorous evaluations of the curriculum among broader populations, both health professions trainees and current healthcare providers, including an assessment of impact on clinical practice and patient care. In addition, movements to integrate understandings of the wider social determinants of health, the unique intersections that influence an individual’s health, and a more comprehensive understanding of what constitutes valid knowledge in healthcare education need to be initiated and supported. Educational initiatives that centre lived experiences can be impactful but cannot be genuinely transformative until there are changes in the medical and medical education systems.

## Data Availability

The data supporting this study's findings are available from the corresponding author upon reasonable request.

## Appendix 2

**Table 4:** Clinicians’ perceptions of usefulness of information delivery format and length of episodes

	*I found the way that the information was delivered to be useful for learning about people’s experiences with weight bias and stigma*					*I found the length of the podcast was appropriate*				
Episode #	Strongly agree	Agree	Neither agree nor disagree	Disagree	Strongly disagree	Strongly agree	Agree	Neither agree nor disagree	Disagree	Strongly disagree
1: Introduction to the podcast 8 min; *n* = 19	37%	53%	5%	5%	0	53%	37%	10%	0	0
2: Introductions to speakers and language 10.5 min; *n* = 19	37%	47%	16%	0	0	32%	47%	16%	5%	0
3: Lived experiences I 11 min; *n* = 19	63%	37%	0	0	0	58%	32%	10%	0	0
4: Lived experiences II 12 min; *n* = 17	38%	56%	6%	0	0	35%	41%	12%	12%	0
5: Health impacts 10 min; *n* = 16	44%	50%	6%	0	0	31%	80%	13%	6%	0
6: Distressed and disordered eating 21 min; *n* = 15	40%	53%	7%	0	0	27%	40%	13%	20%	0
7: How can clinicians do better? 14 min; *n* = 15	47%	53%	0	0	0	27%	40%	27%	6%	0

## Abbreviations

BMI: Body mass index
IBH: Institute for Better Health
LEE: Lived experience education
THP: Trillium Health Partners
